# Sublethal Sodium Hypochlorite Exposure: Impact on Resistance-Nodulation-Cell Division Efflux Pump Overexpression and Cross-Resistance to Imipenem

**DOI:** 10.3390/antibiotics13090828

**Published:** 2024-09-01

**Authors:** Ji-Hyun Nam, Jung Sik Yoo

**Affiliations:** Division of Antimicrobial Resistance Research, National Institute of Infectious Disease, National Institute of Health, Korea Disease Control and Prevention Agency, 187 Osongsaengmyeong2-ro, Osong-eup, Heungdeok-gu, Cheongju-si 28159, Republic of Korea; jungsiku@korea.kr

**Keywords:** cross-resistance, imipenem antibiotics, NaOCl disinfectant, resistance-nodulation-cell division (RND) efflux pump

## Abstract

Sodium hypochlorite (NaOCl) is widely used in public healthcare facilities; this exposure can result in the development of bacterial tolerance to disinfectants, which has known links to antibiotic cross-resistance. However, the mechanism through which cross-resistance to antibiotics and disinfectants develops remains ambiguous. Therefore, this study aimed to examine the phenotypic and transcriptomic changes caused by disinfectant exposure in Gram-negative bacteria and determine the cause of cross-resistance to antibiotics. The results demonstrated that the misuse of disinfectants plays an important role in the emergence of disinfectant resistance and in the increase in antibiotic resistance. Antibiotic resistance may occur from the exposure of Gram-negative bacteria to subminimal inhibitory concentrations (MICs) of NaOCl. Ten passages of Gram-negative bacteria in increasingly higher subMICs of the NaOCl disinfectant were sufficient to increase the MIC to >2500 µg/mL NaOCl, particularly in *K. pneumoniae* and *P. aeruginosa*. To determine the development of cross-resistance to antibiotics due to NaOCl exposure, the MICs for each antibiotic before and after the exposure of each strain to sublethal concentrations of NaOCl were compared. After overnight incubation with a sublethal concentration of NaOCl, a statistically significant increase in MIC was only observed for imipenem (*p* < 0.01). An investigation of the mechanism of cross-resistance by means of transcriptome analysis revealed that 1250 µg/mL of NaOCl-adapted *K. pneumoniae* and *P. aeruginosa* strains increased resistance to imipenem due to the increased expression of resistance-nodulation-cell division (RND) efflux pumps, such as AcrAB-TolC and MexAB/XY-OprM. Therefore, we suggest that exposure to NaOCl can influence the expression of RND efflux pump genes, contributing to imipenem cross-resistance.

## 1. Introduction

Due to the COVID-19 pandemic from 2020 to 2022, the use of disinfectants has increased markedly. The most commonly used disinfectants for combating COVID-19 include quaternary ammonium compounds (QACs), sodium hypochlorite (NaOCl), hydrogen peroxide (H_2_O_2_), and ethanol [1]. The use of disinfectants has rapidly increased worldwide and has been associated with the accelerated emergence of antimicrobial resistance (AMR) in pathogenic microbes in the COVID-19 endemic era starting from 2023 [2,3,4]. The biocide tolerance and antibiotic resistance of one common mechanism exhibited by a certain microorganism can be attributed to cross-resistance between biocides and antibiotics [5]. In general, if a microorganism is intrinsically tolerant to certain QACs or acquires such tolerance after exposure, it is likely to exhibit cross-tolerance to various antimicrobial agents [3,6]. Among the 654 disinfectants listed by the Environmental Protection Agency of the USA for use against severe acute respiratory syndrome virus-2, QACs comprise 45.1%, and chlorines (NaOCl and HOCl) comprise 17.3% of the active ingredients.

Chlorine-based compounds, particularly sodium hypochlorite (NaOCl) or household bleach (4–5%), are among the most frequently used disinfectants. NaOCl is commonly used in healthcare facilities across various settings at concentrations of 0.01% to 0.5% for the spot disinfection of surfaces such as countertops, floors, beds, and other surfaces to eliminate bacterial and viral contaminants (refer to the WHO and ECDC guidelines). Chlorination is widely used for the disinfection of water or wastewater to remove pathogens and potentially antibiotic-resistant bacteria [7]. This general use of chlorine products is attributable to several factors: their broad-spectrum antimicrobial activity, absence of toxic residues, effectiveness even when used with hard water, cost-effectiveness, rapid action, and ability to remove dried or fixed organisms and biofilms from surfaces [8]. The exact mechanism by which free chlorine destroys microorganisms has not been elucidated; however, several factors have been proposed, including the oxidation of intracellular content, the inhibition of protein synthesis, decreased oxygen uptake, the oxidation of respiratory components, decreased ATP production, breaks in DNA, and the inhibition of DNA synthesis [9]. However, commonly used chlorine disinfectants are less effective against highly chlorine-resistant waterborne bacteria, such as pathogenic *Pseudomonas aeruginosa*, which can exist in drinking tap water and resist disinfection.

Several shared resistance mechanisms have been reported for disinfectants and antibiotics [10,11,12]. The cross-resistance between antibiotics and disinfectants may occur via cellular mechanisms that protect against multiple classes of antimicrobial agents or via the selection of genetic determinants for resistance to non-antibiotic agents that are linked to genes for antibiotic resistance [9]. Previous studies have shown that many proteobacterial species that are considered to be critical priority AMR pathogens (e.g., *Enterobacteriales*, *Acinetobacter* spp., and *Pseudomonas* spp.) are intrinsically tolerant to higher concentrations of chlorine [13,14]. These species have demonstrated an ability to adapt to NaOCl upon prolonged or repeated exposure to sublethal NaOCl concentrations, ultimately leading to increased disinfectant tolerance, biocide cross-tolerance, and cross-resistance to clinically relevant antibiotics [2]. The fact that the annual global use of disinfectants is more prevalent than that of antibiotics remains a major concern [13,15]. Such overuse of disinfectants has made these compounds common pollutants in ecosystems.

In Gram-negative bacteria, the most clinically relevant efflux pumps are resistance–nodulation–division (RND) family members, which recognize a broad range of substrates, including antibiotics and disinfectants such as QACs/chlorhexidine [16]. This family includes well-characterized members of the Enterobacteriaceae multidrug-resistant (MDR) efflux pumps AcrAB-TolC and MexAB-OprM from *Pseudomonas aeruginosa* and AdeABC in *Acinetobacter baumannii*. These strains often demonstrate the upregulation of MDR efflux pumps, such as AcrAB-TolC. While efflux is linked to increased biocide tolerance, little is known about the contributions of these individual efflux pumps to biocide tolerance.

Due to society’s reliance on and overuse of disinfectants, understanding how sodium hypochlorite (NaOCl) may drive antimicrobial resistance is crucial. Sustained exposure to sublethal levels of disinfectants can lead to MDR; however, the mechanism through which cross-resistance to antibiotics and disinfectants develops remains ambiguous. Therefore, we hypothesized that disinfectant-induced tolerance mechanisms (i.e., specific efflux pumps) to antibiotic cross-resistance could be more clearly elucidated by transcriptomic analysis. Therefore, this study tested the phenotypic and transcriptomic changes caused by disinfectant exposure in Gram-negative bacteria to determine the cause of cross-resistance to antibiotics.

## 2. Results

### 2.1. Effect of Exposure to Disinfectants on Gram-Negative Bacteria

The MICs for NaOCl in 121 wild-type isolates of Gram-negative bacteria were determined using the broth microdilution method. The NaOCl disinfectant MIC values of *Enterobacteriaceae*, including carbapenem-resistant *A. baumannii*, ranged from 250 to 500 µg/mL and 250–1000 µg/mL, respectively. In particular, the MICs for *P. aeruginosa* strains were above the median of other species and ranged from 250 to 1000 µg/mL of NaOCl.

Among the 121 strains used (including reference strains), the MIC for NaOCl of 10 strains increased by a fold change ≥ 2.0 after NaOCl exposure (Table 1). All *E. coli* and *A. baumannii* isolates showed a non-adaptive response (MIC increase < 2-fold). A strong and stable MIC change was observed for isolates of *K. pneumoniae* and *P. aeruginosa*. In the control experiment (passages without disinfectant exposure), no significant changes in MICs were observed. As shown in Table 1, 10 passages of Gram-negative bacteria cultured in gradually higher subMICs of the disinfectant were sufficient to increase the MIC for NaOCl up to 2500 µg/mL. The stability of NaOCl-adapted strains was tested in the absence of each disinfectant over five additional passages. The MIC of NaOCl was again tested at the end of these passages, and this value was still higher than the initial value.

### 2.2. Antibiotic Susceptibility Was Reduced According to NaOCl Exposure

The MIC values before and after exposure to disinfectants were compared using the Mann–Whitney U test to determine the effect of the overnight incubation of Gram-negative bacteria with 1250 μg/mL of NaOCl on the MICs of antibiotics. As shown in Table 1, in isolates adapted to NaOCl, a statistically significant increase in MIC was observed for imipenem (*p* = 0.010, 83.3% of all NaOCl-adapted strains). Particularly in the case of *Enterobacteriaceae*, NaOCl significantly increased the MIC for imipenem only (*p* = 0.006). We did not observe any changes in the MIC of *P. aeruginosa* with high levels of carbapenem resistance for imipenem.

### 2.3. Cross-Resistance between NaOCl Disinfectant and β-Lactams in Klebsiella pneumoniae

The KEGG pathway analysis was performed to explore the biological functions and pathways of DEGs. In the KEGG analysis of adapted and wild-type *K. pneumoniae* Z0318KP0159, 951 DEGs were identified and assigned to 93 pathways. Between adapted and wild-type *K. pneumoniae* Z0318KP0107, 1472 DEGs in 110 pathways were identified. The significantly upregulated gene-enriched pathways included the two-component system (Bonferroni corrected *p* = 7.7 × 10^−16^), phosphotransferase system (PTS, Bonferroni corrected *p* = 1.6 × 10^−16^), and oxidative phosphorylation (Bonferroni corrected *p* = 9.2 × 10^−9^), which included energy metabolism (Figure S1). The most notable result involved the upregulation genes annotated in the “β-lactam-resistance” pathway (Bonferroni corrected *p* = 0.01). The β-lactam-resistance pathway included 8 and 10 DEGs in Z0317KP0159 and Z0318KP0107, respectively (Figure S1).

The overexpression of RND efflux pump genes in *K. pneumoniae* Z0317KP0159 and Z0318KP0107 after exposure to 1250 μg/mL NaOCl promoted resistance to β-lactams (Table 2). The expression of AcrAB-TolC genes, associated with the RND efflux pump, was increased by at least two-fold, except for tolC in *K. pneumoniae* Z0318KP0107. Moreover, the β-lactam-resistance pathway of Z0317KP0107 was significantly upregulated (*p* < 0.001). The treatment of *K. pneumoniae* Z0318KP0107 with NaOCl-induced overexpression of the MarRAB operon (67.7-fold increase in *marA* expression), a global antibiotic-resistance regulator involved in the production of the AcrAB-TolC efflux pump that extrudes antibiotics. As shown in Table 1, antibiotic phenotypic characteristics after exposure to 1250 μg/mL NaOCl also significantly reduced susceptibility to imipenem using the Mann–Whitney U test (*p* = 0.006). This implies that increased resistance to β-lactam antibiotics (including carbapenem) and the overexpression of AcrAB-TolC are associated with NaOCl exposure. In *K. pneumoniae*, β-lactamase, and carbapenemase genes showed strain-specific differences in expression after NaOCl exposure.

### 2.4. Cross-Resistance between NaOCl Disinfectant and β-Lactams in Pseudomonas aeruginosa

In the KEGG pathway analysis of adapted and wild-type *P. aeruginosa* ATCC 27853, 1328 DEGs were identified and assigned to 107 pathways, while 1332 DEGs, assigned to 110 pathways, were identified in *P. aeruginosa* Z0219PA0007, and 1309 DEGs assigned to 104 pathways were identified in *P. aeruginosa* Z0217PA0020. As shown in Figure S2, the expression of genes was significantly related to biofilm formation in *P. aeruginosa* Z0219PA0007, in which the upregulated gene-enriched pathways were associated with the two-component system (Bonferroni corrected *p* = 2.3 × 10^−25^), biofilm formation (Bonferroni corrected *p* = 2.2 × 10^−23^), the bacterial secretion system (Bonferroni corrected *p* = 8.2 × 10^−23^), and quorum sensing (Bonferroni corrected *p* = 5.5 × 10^−5^). Likewise, the most notable change in *Pseudomonas*, as shown in Figure S2, was the upregulation of the pathway related to β-lactam-resistance (Bonferroni corrected *p* = 9.7 × 10^−4^ in *P. aeruginosa* Z0219PA0007). β-lactam-resistance was associated with many DEGs as follows: ATCC 27853, Z0219PA0007, and Z0217PA0020 showed the upregulation of 12, 16, and 17 DEGs, respectively.

The overexpression of the RND efflux pump genes in *P. aeruginosa* ATCC 27853, Z2019PA0007, and Z0217PA0020 after exposure to 1250 μg/mL of NaOCl also promoted resistance to β-lactam antibiotics (Table 3). The expression of MexAB-XY efflux pump genes associated with the RND efflux pump was increased by at least two-fold, except for mexAB-oprM in *P. aeruginosa* ATCC 27853. As shown in Figure S2, the β-lactam-resistance pathways of Z2019PA0007 and Z0217PA0020 were significantly upregulated (*p* < 0.001). In particular, the NaOCl treatment of the three adapted *P. aeruginosa* strains commonly induced the overexpression of *armZ-mexXY-oprM* (min 2.09 vs. max. 21.02-fold increase), which encodes a global antibiotic-resistance regulator/modulator involved in the production of the MexXY-OprM efflux pump that extrudes antibiotics. In *P. aeruginosa* ATCC 27853, MexEF-OprN efflux pump gene expressions increased by at least 2-fold, whereas the expression of ParRS regulator genes decreased.

NaOCl exposure (adaptation) in *P. aeruginosa* also altered the gene expression related to the AmpC-AmpR-AmpG pathway. However, AmpC overexpression for β-lactamase production was not observed in this study. The genes associated with β-lactamase resistance in *P. aeruginosa* also exhibited strain-specific differential expression upon NaOCl exposure. This confirmed that increased resistance to β-lactams (including carbapenem) and the overexpression of the RND efflux pump are linked to NaOCl tolerance in *P. aeruginosa* and *K. pneumoniae*.

## 3. Discussion

Household items that contain disinfectants may be used “inadequately” by consumers, and diluted products and/or residues may allow for the growth of multidrug efflux pump hyper-expressing strains that are concomitantly multidrug-resistant, which may pose a pressing epidemiological issue. Processes that are demonstrated in a laboratory may also be reproduced by humans and in the environment. Thus, in this study, the initial MIC values of unadapted strains for disinfectants were compared with the MIC values of NaOCl-adapted strains. As shown in Table 1, NaOCl-adapted Gram-negative bacteria were not killed by sublethal or recommended disinfectant concentrations (500–5000 μg/mL). We demonstrated that 10 passages of Gram-negative bacteria in increasingly higher sublethal MICs of NaOCl disinfectant were sufficient to increase the MIC for NaOCl to >2500 µg/mL, particularly in *K. pneumoniae* and *P. aeruginosa*. Moreover, the MICs of each of the tested strains for a range of antibiotics before and after exposure to sublethal concentrations of NaOCl were compared. A statistically significant increase in MIC was only observed for imipenem (*p* < 0.010). In a previous study, 5000 μg/mL of NaOCl showed a lethal effect on 94.1% of *P. aeruginosa* isolates [17]. Ni et al. recommended that disinfectant concentrations of chorine-containing disinfectants for carbapenem-resistant *K. pneumoniae* (CRKP) disinfection be set at 2000–5000 μg/mL in China [18]. In addition, Kanamori et al. demonstrated that disinfectants commonly used in healthcare facilities could likely be effective (>3log_10_ reduction) against carbapenem/colistin-resistant *Enterobacteriaceae* when used at appropriate concentrations, such as ≥5000 μg/mL NaOCl [19]. These results indicate that NaOCl should not be used at sublethal concentrations in order to lower the risk of developing bacterial tolerance and resistance to antibiotics.

Whole genome sequencing (WGS) was conducted to elucidate the gene differences among wild and NaOCl-adapted *K. pneumoniae* and *P. aeruginosa* strains, respectively. The average nucleotide identity (OrthoANI) analysis of the draft genomes revealed a similarity of over 99.9%, suggesting that wild-type and NaOCl-adapted strains of *K. pneumoniae* and *P. aeruginosa* are essentially identical (for more details, please refer to Table S1 in the Supplementary Materials). The analysis identified mutations in the coding regions of several hypothetical proteins and partial ribosomal RNA between wild-type and NaOCl-adapted strains. However, transcriptome analysis revealed that 1250 µg/mL of NaOCl-adapted *K. pneumoniae* and *P. aeruginosa* strains increased resistance to imipenem due to increased expression of the RND superfamily efflux pumps, such as AcrAB-TolC and MexAB/XY-OprM. In the case of NaOCl exposure, only the *bla*_KPC-2_ gene was overexpressed among the β-lactam resistance genes on the plasmid of *K. pneumoniae* Z0318KP0107 (Table 2). In addition, all wild and adaptive strains of *Pseudomonas* did not contain any plasmid. Therefore, our data might mean that NaOCl results in cross-resistance more than co-resistance with antibiotics (β-lactams).

Bacterial efflux pumps with inherent/acquired biocide tolerance can reduce susceptibility to other biocides and induce cross-resistance to specific antibiotics [12]. On the contrary, mechanisms of tolerance to biocides, such as permeability, degradation, and mutation, can also result in an increase in tolerance or lead to cross-resistance. This report also concludes that increased resistance to other biocides and cross-resistance to certain antibiotics is possible if phenotypic changes and induction occur due to biocide exposure. This study aimed to investigate the tolerance of Gram-negative bacteria isolated from humans and the environment to NaOCl and evaluate cross-resistance to antibiotics after exposure to this disinfectant. Adaptation and tolerance to QACs and chlorhexidine are attributed to the presence and upregulation of specific efflux pumps, such as the small multidrug resistance pump [2,20], while NaOCl induces the expression of many functional gene families, including those associated with responses to oxidative stress, DNA repair, energy metabolism, membrane damage, and efflux pumps [21].

In particular, the RND family of efflux pumps strongly promotes inherent antibiotic-resistant Gram-negative bacteria. The components of the RND efflux pump include an inner membrane pump protein specific to a particular substrate, an outer membrane protein, and a periplasmic accessory protein that binds to both the inner and outer membrane proteins, allowing the extrusion of substrates from the cell. AcrAB-TolC and MexAB-OprM are the major RND efflux systems present in *E. coli* and *P. aeruginosa* and are essential for their survival, colonization, and virulence [22]. In the present study, we revealed the effect of NaOCl disinfectant on the promotion of microbial tolerance to disinfectant and antibiotic resistance in *K. pneumoniae* and *P. aeruginosa*.

**Figure 1:**
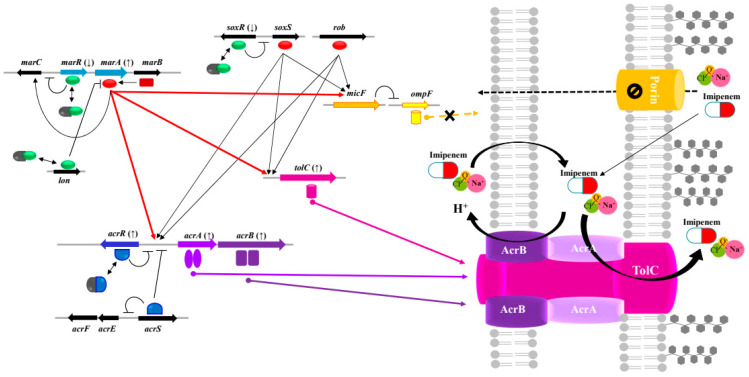
Acquisition of cross-resistance to imipenem antibiotic and NaOCl disinfectant by ArcAB-TolC efflux pump-related gene expression in NaOCl-exposed *Klebsiella pneumoniae*. Gene expression prediction based on RNA-Seq and quantitative reverse-transcription polymerase chain reaction datasets of *K. pneumoniae* strains (Z0318KP0107 and Z0317KP0159). The depiction of the AcrAB-TolC efflux system was modified from the work of Li et al., Jia et al., and Li et al. [23,24,29]. AcrAB-TolC is a tripartite complex formed by AcrA, a membrane fusion protein AcrB, a cytoplasmic-membrane protein, and TolC, an outer membrane protein. *acrA* and *acrB* are part of the same operon, which is negatively regulated by the local repressor (blue semicircle) AcrR. The genes are represented as arrows, and their translated proteins are represented as green ovals (repressors) and red ovals (activators). The activation of *acrAB-tolC* and *micF* transcription occurred primarily because of the global regulatory proteins MarA, SoxS, and Rob (red ovals). The arrows in parenthesis represent the down/upregulation of gene expression. The bold red arrowed lines indicate that MarA increased its own transcription and activated the expression of *acrAB-tolC* and *micF* due to NaOCl exposure in our study. Overall, under conditions of NaOCl exposure, these multiple regulation mechanisms can create cross-resistance by simultaneously allowing both the decreased influx (via OmpF porin) and increased efflux (via AcrAB-TolC) of imipenem antibiotics.

Among the RND pumps in *Enterobacteriaceae*, AcrAB-TolC is the most clinically important antibiotic efflux pump [23]. Our results, summarized in Figure 1 and Figure S1 and Table 1, Table 2 and Table S2, show that 1250 μg/mL of NaOCl-adapted *K. pneumoniae* Z0317KP0159 and Z0317KP0107 show increased resistance to β-lactam antibiotics (particularly imipenem) due to the increased expression of the AcrAB-TolC efflux pump system. The AcrAB-TolC efflux pump has three global regulators: MarA, SoxS, and Rob [23]. Multiple regulators play important roles in promoting the expression of *acrA/B*, *tolC*, and *micF*, which are genes in the *marA-soxS-rob* regulon. The *micF* transcript inhibits the translation of *ompF* porin mRNA, which provides an entry channel for small hydrophilic antibiotics (β-lactams, aminoglycosides, and colistin). The multiple antibiotic-resistance (*mar*) locus mediates resistance primarily by upregulating the efflux of some antibiotics, disinfectants, and organic solvents via the AcrAB-TolC efflux pump and downregulating influx through the outer membrane protein F [24]. Encoded by a mar locus containing *marR/A/B*, MarA positively regulates the expression of *marR/A/B* and many other genes (*acrA/B*, *tolC*, *micF*, etc.). SoxS and the Rob activator also stimulate the expression of many genes under the mar regulon. In addition, SoxR is activated by superoxide compounds, such as NaOCl and H_2_O_2_ [11]. The oxidation of SoxR induces the activation of a second redox sensor, SoxS, which induces the transcription of several genes (manganese superoxide dismutase, ferredoxin, *micF*, etc.).

We confirmed that AcrAB-TolC efflux pump-related genes were overexpressed in 1250 μg/mL NaOCl-adapted *K. pneumoniae* using qRT-PCR (Table S2). In particular, the regulatory gene *marR* showed decreased expression, whereas the expression of the positive regulatory gene *marA*, which serves downstream of these regulators, was increased. Consequently, the expression of genes encoding multidrug efflux pumps (*acrA/B*, *tolC*) was increased. In contrast, expression levels of *acrR* and *soxR* were upregulated or had different regulatory levels, depending on the strain. Therefore, we concluded that the overexpression of *marA* in *K. pneumoniae* strains made the adapted strains less sensitive to the effects of NaOCl. Our results also confirm that the overexpression of *marRAB* led to cross-resistance between NaOCl and imipenem. These results are similar to the previous hypothesis by Randall and Woodward and Chetri et al. [25,26]. Although the level of antibiotic resistance conferred by *marRAB* was relatively low, increasing evidence suggests that *marRAB* and related systems are important for clinical antibiotic resistance, likely serving as a ‘stepping stone’ to achieve higher levels of resistance, such as those of carbapenems.

Under various conditions, these multiple regulatory mechanisms can induce cross-resistance to NaOCl and imipenem by allowing the simultaneously decreased influx (via the OmpF porin) and increased efflux (via AcrAB-TolC) of antimicrobial agents. Recent studies have suggested that chlorhexidine-adapted strains of *K. pneumoniae* are cross-resistant to other biocides and antibiotics, presumably because the upregulation of *acrAB* and *ramA,* in turn, activate the AcrAB-TolC efflux pump [27,28]. In both these studies, the activation of AcrAB-TolC resulted in the reduced susceptibility of *K. pneumoniae* to several antibiotics and biocides, including chlorhexidine, triclosan, and QACs. This result is consistent with our findings. In summary, we hypothesized that NaOCl exposure would influence gene expression, particularly those related to the AcrAB-TolC efflux pump of the RND family in *K. pneumoniae*, contributing to imipenem resistance. Based on this hypothesis, we described the gene expression levels of NaOCl–imipenem cross-resistance involving (1) a regulator (especially MarA), (2) a drug transporter and efflux pump, (3) the cell membrane structure and transporter protein, and (4) loss of porin, as shown in Figure 1.

**Figure 2:**
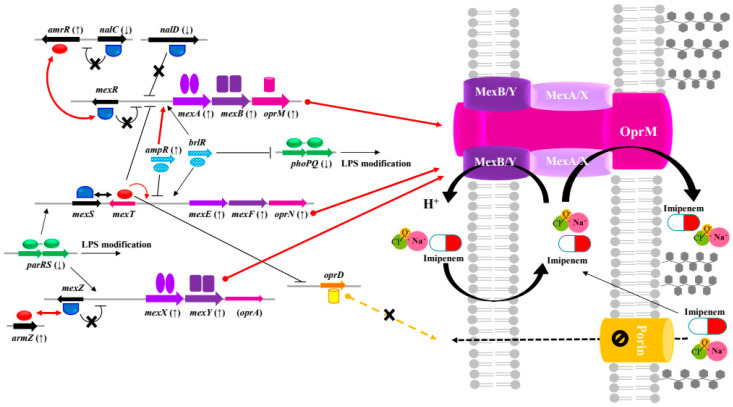
Induction of cross-resistance to imipenem antibiotics by the overexpression of the Mex-Opr efflux pump system in NaOCl-exposed *Pseudomonas aeruginosa*. Gene expression prediction was based on the transcriptome analysis of *P. aeruginosa* (ATCC 27853, Z0219PA0007, and Z0217PA0020). The depiction of the efflux system was modified from the work of Li et al. and Moradali et al. [23,29,33]. The genes are represented as arrows, and their translated proteins are represented as blue semicircles (repressors) and red ovals (activators). The arrows in parenthesis represent the down/upregulation of gene expression. The bold red arrowed lines indicate that AmrR and ArmZ increase their own transcription and activate the expression of *mexAB/XY-oprM* upon NaOCl exposure. Overall, under conditions of NaOCl exposure, these multiple regulation mechanisms can create cross-resistance by simultaneously allowing the increased efflux of imipenem antibiotics.

Among the RND pumps in *P. aeruginosa*, the most clinically important antibiotic efflux pumps are MexAB-OprM, MexXY-OprM, MexCD-OprJ, and MexEF-OprN [23]. Verdial et al. described the overexpression of RND efflux pump systems as a common intrinsic or acquired resistance trait in *P. aeruginosa* [30]. They reported on the overexpression of MexAB-OprM and MexXY-OprM results in *P. aeruginosa* resistance to aminoglycosides and β-lactams and that *mexAB-oprM*, *mexCD-oprJ*, and *mexEF-oprN* are among the most studied gene-encoding regulators of QACs, chlorhexidine, and trichlosan tolerance. As summarized in Figure 2 and Table 1 and Table 3, our results show that NaOCl-adapted *P. aeruginosa* Z0219PA0007 and Z0217PA0020 have reduced sensitivity to imipenem due to the increased expression of MexAB-OprM and MexXY-OprM efflux pump systems. In the case of 1250 μg/mL of NaOCl-adapted *P. aeruginosa* ATCC 27853, decreased sensitivities to β-lactam antibiotics were due to an increase in the expression MexEF-OprN and MexXY-OprM. Hou et al. showed that chlorine-injured *P. aeruginosa* cells that were exposed to sublethal concentrations (4 μg/mL) of NaOCl developed increased resistance by 1.4–5.6-fold to ceftazidime, ampicillin, and chloramphenicol [28]. These results were confirmed by quantitative PCR, which showed that genes related to the MexEF-OprN efflux pump were overexpressed. Bubonja-Sonje et al. revealed that approximately 30% of 62 isolates (mostly obtained from intensive care unit patients with reduced carbapenem susceptibility) showed an increased production of transcripts related to MexEF-OprN (from 4- to 19-fold in *mexF* mRNA transcripts compared with a wild-type reference isolate) [31]. Li et al. reported that the MexEF-OprN efflux system was not well-expressed in wild-type *P. aeruginosa*, and thus, its inactivation led to little or no change in antibiotic susceptibility [23]. In *P. aeruginosa*, where even small antibiotics must slowly diffuse across the outer membrane (OM) via slow porin, the active efflux of the major RND pump is very effective in increasing the MICs of antibiotics [29]. In addition, imipenem can penetrate the OM much more rapidly than can other antibiotics by utilizing a specific channel, OprD. Dulyayangkul et al. reported that hypochlorite triggers the overexpression of major facilitator superfamily (MFS) pumps in *Pseudomonas aeruginosa* [32]. They also reported that increasing the production of MexXY mediated by ArmZ reduces antibiotic susceptibility. Our results did not indicate the loss of OprD or other porin proteins related to porin transcription and β-lactam-resistance (Table 3). Based on our results, we suggest that the MexXY-OprM efflux pump of the RND family is involved in cross-resistance to NaOCl and imipenem. In addition, as shown in Figure 2, NaOCl and imipenem cross-resistance involved local regulators and gene expression related to the RND (MexAB, MexXY, and MexEF) efflux pumps of *P. aeruginosa*. Taken together, our results suggest that NaOCl disinfectant exposure influences the expression of genes that contribute to imipenem cross-resistance, particularly those related to the RND efflux pump in Gram-negative bacteria.

## 4. Materials and Methods

### 4.1. Isolation and Identification of Bacterial Strains

Overall, 117 bacterial strains were included in this study (Table S3). Of them, 91 isolates were obtained from humans, including 41 *Escherichia coli*, 9 *Klebsiella pneumoniae*, 26 *A. baumannii*, and 15 *P. aeruginosa strains*. Furthermore, 26 isolates were obtained from hospital and livestock environments: 7 *E. coli*, 4 *K. pneumoniae*, 11 *A. baumannii*, and 4 *P. aeruginosa*. All isolates from humans were MDR, including resistance to carbapenems. Environmentally obtained strains of *K. pneumoniae* and *A. baumannii* are known to be MDR, including resistance to carbapenems. The standard strains included *E. coli* ATCC 25922 and ATCC 10536, *A. baumannii* ATCC 19606, and *P. aeruginosa* ATCC 27853.

### 4.2. Determination of Antibiotic and Disinfectant Minimum Inhibitory Concentrations (MICs)

Antimicrobial susceptibility testing was performed using the broth microdilution method with a customized Sensititre KRCDC2F panel (TREK Diagnostic Systems, East Grinstead, United Kingdom) in accordance with the guidelines established by the Clinical and Laboratory Standards Institute [34]. The following antimicrobial agents were tested: amikacin, ampicillin, azithromycin, cefotaxime, cefoxitin, ceftazidime, ceftriaxone, chloramphenicol, ciprofloxacin, colistin, imipenem, gentamicin, nalidixic acid, streptomycin, tetracycline, and trimethoprim/sulfamethoxazole. All experiments in this study were carried out with independent biological replicates at least 3 times.

For NaOCl, household chlorine bleach (Clorox ≥ 4%, Yuhan Corp, Seoul, Republic of Korea) was used. To determine the initial exposure concentrations of the disinfectant, the MIC of NaOCl was tested. Briefly, an overnight bacterial culture was diluted with a sterile 0.85% NaCl solution to a 0.5 McFarland standard. Then, 96-well plates were prepared, and 90 μL of bacterial suspension (1.5 × 10^6^ CFU/mL) containing 10 μL of a serially, two-fold diluted NaOCl disinfectant in a cation-adjusted Mueller–Hinton broth (CA-MHB) medium, was added to each well. For the growth control group, 90 μL of bacterial suspension was added to 10 μL of MHB medium without NaOCl disinfectant. For the negative control group, 90 μL of a sterile 0.85% NaCl solution was used instead of bacterial suspension.

### 4.3. NaOCl Disinfectant Exposure and Bacterial Subculturing

To determine their adaptation to the disinfectant, strains were exposed to increasing sublethal concentrations of NaOCl. Each disinfectant concentration was continuously increased two-fold, and the cells were subcultured for 2 weeks using 96-well plates. Specifically, 10 μL of the overnight culture of wild-type Gram-negative bacteria was first added to each of at least eight wells containing 90 μL of the CA-MHB medium with 2× sublethal concentration of NaOCl. These plates were incubated at 37 °C with shaking at 150 rpm for 24 h. When growth was observed at the highest inhibitory concentration, this culture suspension was inoculated into a well-containing medium with a NaOCl concentration two-fold higher than the previous concentration. Finally, we selected disinfectant-adapted strains that increased the disinfectant MIC of the wild-type strain by at least two-fold and maintained the highest MICs for 10 continuous subcultures.

### 4.4. RNA Extraction, Transcriptome Sequencing, and Quantitative Reverse Transcription Polymerase Chain Reaction

Wild-type strains were cultured in CA-MHB for 16–18 h at 37 °C. Adapted isolates were cultured in CA-MHB supplemented with 1250 μg/mL of NaOCl for 24 h at 37 °C. The adapted strains included *K. pneumoniae* Z0317KP0159, *K. pneumoniae* Z0318KP0107, *P. aeruginosa* ATCC 27853, *P. aeruginosa* Z2017PA0020, and *P. aeruginosa* Z2019PA0007. Cell pellets derived from exponential phase cultures of each bacterial strain were stored at −80 °C in a 10× volume of the stabilization reagent RNALater (ThermoFisher Scientific, Waltham, MA, USA). DNA and RNA were extracted using the methods described and sequenced below.

Total RNA was extracted using the TRIzol reagent (Invitrogen, Waltham, MA, USA) according to the manufacturer’s protocol. RNA samples were purified using the RNeasy Mini Kit (Qiagen, Munchen, Germany), including on-column DNase digestion, according to the manufacturer’s protocol. RNA purity was assessed using a NanoDrop spectrophotometer (Thermo Fisher Scientific, Waltham, MA, USA). The RNA concentration was measured using a Qubit RNA BR Assay Kit and a Qubit 4 Fluorometer (Invitrogen). The extracted RNA was used for strand-specific cDNA library construction and Illumina paired-end sequencing (HiSeq 2500; Illumina Inc., San Diego, CA, USA) at Macrogen Co. (Seoul, Republic of Korea). mRNA expression levels were normalized to fragments per kb of transcript per million mapped reads (FPKM). The database analysis of Annotation, Visualization, and Integrated Discovery (DAVID) used edgeR to identify genes that were differentially expressed (DEGs) between the wild-type and NaOCl-adapted strains. Potential targets of the DEGs were analyzed using the Gene Ontology term (GOTERM) and Kyoto Encyclopedia of Genes and Genomes (KEGG) pathway maps.

AcrAB-TolC pump-related gene expression (regulator/transporter) was quantified by the modification of the quantitative reverse transcription polymerase chain reaction (qRT-PCR) method described by Jia et al. [26]. A significant effect on gene expression was deduced when the corresponding ratios exceeded 2.0. All reactions were performed in triplicate.

### 4.5. Statistical Analysis

The statistical significance of MIC data (disinfectant-wild versus -adapted strain upon exposure to a specific antibiotic) was evaluated using the nonparametric Mann–Whitney U test. The experiment was repeated at least 5 times. Significance was set at *p* < 0.05. KEGG enrichment analysis of the disinfectant-adapted strain was performed using Fisher’s exact test with the Bonferroni correction (*p* < 0.05). All statistical calculations were performed using SPSS version 24 (IBM Corp., Armonk, NY, USA).

## 5. Conclusions

Disinfectants have seen a rapid increase in use, particularly in recent years, due to the ongoing COVID-19 pandemic. The most used disinfectants for combatting COVID-19 include NaOCl, QACs, H_2_O_2_, and ethanol. The mode of action of these disinfectants, except for triclosan, which has a single specific target, is nonspecific. The mechanism of resistance to disinfectants involves several transcriptional regulators, and no single gene has been linked to NaOCl resistance. NaOCl often leads to the overexpression of the efflux pump, which may confer resistance to multiple antimicrobials. Although RND efflux pumps are overexpressed in the presence of NaOCl, they are associated with increased cross-resistance to some β-lactam antibiotics (carbapenems, particularly imipenem) and other disinfectants.

In general, if recommended guidelines for the use of disinfectants are followed in a way that limits the exposure of bacteria to sublethal doses of disinfectants, the risk of developing resistance and cross-resistance could be eliminated. Therefore, the use of disinfectants that carry a high risk of antimicrobial resistance, such as NaOCl and QACs, in household products and over-the-counter medications should be reevaluated. The use of disinfectants has increased substantially since the onset of the COVID-19 pandemic. Our findings highlight a need to monitor the cross-resistance between different disinfectants (e.g., chlorhexidine, H_2_O_2_) and clinically important antibiotics (e.g., tigecycline, colistin). Additionally, the findings emphasize the importance of disinfectants in the development and spread of antibiotic-resistant bacteria.

## Figures and Tables

**Table 1:** Evaluation of NaOCl minimal inhibitory concentration and antibiotic susceptibility changes with disinfectant exposure.

Species		Strains	MICs (mg/L)		Antibiotic Susceptibility (Exposure to 1250 µg/mL NaOCl)	
			Before ^1^	After	Increased	Reduced
*K. pneumoniae*	CRKP *	Z0317KP0159	500	2500	FOX, IMI, CHL ^†^	ND ^2^
		Z0317KP0181	500	2500	IMI	GEN ^‡^
		Z0318KP0099	500	2500	IMI	STR
		Z0318KP0107	500	2500	IMI, CHL, AZI	ND
		Z0318KP0236	500	2500	IMI, CHL	GEN, AZI
*P. aeruginosa*	CSPA	ATCC 27853	500	2500	AXO, IMI, STR, COL	TAZ, CIP, NAL, TET, GEN, AMI, SXT
		Z0219PA0007	500	1250	AXO, IMI, COL	CIP, NAL, STR, SXT
	CRPA	I0020PA0021 (HL-IMI)	500	2500	X ^3^	X
		I0020PA0028	500	1250	IMI, CIP, NAL, TET, AMI, STR	FOT, TAZ, AXO, GEN
		Z0217PA0020 (HL-IMI)	500	2500	FOT, TAZ, CIP, CHL, GEN, STR, SXT	NAL, AMI

^1^ “Before” indicates no exposure to disinfectants (wild-type strain), and “After” indicates exposure to disinfectants (adapted-type strains). ^2^ ND: not detected. ^3^ X: exposure to disinfectants does not alter antibiotic susceptibility profiles. * CRKP, carbapenem-resistant *Klebsiella pneumoniae*; CSPA, carbapenem-susceptible *Pseudomonas aeruginosa*; CRPA, carbapenem-resistant *P. aeruginosa*; FOT, cefotaxime; AXO, ceftriaxone; FOX, cefoxitin; TAZ, ceftazidime; AMP, ampicillin; IMI, imipenem; HL-IMI, high-level imipenem resistant; CIP, ciprofloxacin; NAL nalidixic acid; TET, tetracycline, CHL, chloramphenicol; GEN, gentamicin; AMI, amikacin; STR, streptomycin; AZI, azithromycin; COL, colistin; and SXT, trimethoprim/sulfamethoxazole. ^†^ Red words: antibiotic susceptibility profiles changed “susceptible” to “resistant” (S→R). ^‡^ Blue words: antibiotic susceptibility profiles changed “resistant” to “susceptible” (R→S).

**Table 2:** Fold change in the expression of genes related to β-lactam antibiotic resistance in *Klebsiella pneumoniae* after exposure to 1250 μg/mL of NaOCl.

Group	Function	Gene Locus Tag (Gene Name)	Gene Expression Fold Change *	
			Z0317 KP0159	Z0318 KP0107
Enzymatic degradation	Class A carbapenemase Kpc-2	KPHS_p200360 (*bla*_KPC-2_)	−2.26	3.40
	β-lactamase	KPHS_25220 (*bla*_SHV_)	2.43	−1.44
		KPHS_13880	-	2.71
RND efflux pump	Repression of porin OmpF (*mar-sox-rob* regulon activator)	KPHS_25470 *(marA)*	−1.44	67.73
	Multidrug efflux membrane fusion protein	KPHS_11890 (*acrA*)	1.46	5.18
	Multidrug efflux transporter	KPHS_11880 (*acrB*)	2.19	2.30
	Multidrug efflux transporter (permease EefB)	KPHS_52090 (*acrB*)	2.07	2.65
	Outer membrane channel protein	KPHS_45760 (*tolC*)	2.83	1.30
	Multidrug efflux transport outer membrane protein EefC	KPHS_52100 (*adeK*)	2.13	−1.16
Porin	Outer membrane protein 1A/OmpK35 porin	KPHS_18370 (*ompF*)	−2.45	1.52
	Outer membrane pore protein C/OmpK36 porin	KPHS_37010 (*ompC*)	-	2.22

* Difference in fold change to antibiotic-resistance gene expression before and after exposure to 1250 µg/mL of NaOCl.

**Table 3:** Fold change in β-lactam-resistance gene expression in 1250 µg/mL of NaOCl-adapted *Pseudomonas aeruginosa*.

Group	Function		Gene Locus Tag (Gene Name)	Gene Expression Fold Change *		
				ATCC 27853	Z0219 PA0007	Z0217 PA0020
β- lactamase	AmpC beta-lactamase		PA4110 (*ampC*)	-	−2.32	−1.07
	Beta-hexosaminidase		PA3005 (*nagZ*)	4.30	-	-
	Transport of degraded muropeptides (GlcNAc-anhMurNAc)		PA4218 (*ampG*)	−6.95	13.74	1.92
RND efflux pump	MexAB-OprM	MexR anti-repressor ArmR	PA3719 (*armR*)	-	2.29	3.87
		Transcriptional regulator AmpR	PA4109 (*ampR*)	-	2.31	2.62
		Transcriptional regulator	PA3721 (*nalC*)	−2.65	-	-
		Transcriptional regulator	PA3574 (*nalD*)	−2.39	−2.33	−1.75
		Multidrug resistance protein MexA	PA0425 (*mexA*)	-	2.50	2.03
		Multidrug resistance protein MexB	PA0426, PA4375 (*mexB*)	-	2.75	3.09
		Outer membrane protein OprM	PA0427 (*oprM*)	-	2.09	3.06
	MexEF-OprN	Multidrug efflux membrane fusion protein MexE	PA2493 (*mexE*)	11.88	-	-
		Multidrug efflux transporter MexF	PA2494 (*mexF*)	4.11	-	-
		Transcriptional regulator AmpR	PA2495 (*oprN*)	9.75	-	-
	MexXY-(OprM)	Transcriptional regulator	PA2020 (*mexZ*)	-	2.84	−2.21
		Two-component response regulator ParR	PA1799 (*parR*)	−7.73	−2.53	−2.06
		Multidrug efflux membrane fusion protein	PA2019 (*mexX*)	4.04	20.45	8.88
		Multidrug efflux transporter	PA2018 (*mexY*)	2.32	21.02	8.00
		Outer membrane protein	PA4144 (*oprM*)	2.64	1.11	−2.13
		MexZ anti-repressor	PA5471 (*armZ*)	9.59	4.59	10.34
	MexVW-OprM	Multidrug efflux membrane fusion protein MexV	PA4374 (*mexV*)	-	-	-
		Multidrug efflux membrane protein	PA4375 (*mexW*)	-	1.31	2.62
		Outer membrane protein OprM	PA4974 (*oprM*)	-	−2.63	5.65
Porin		Porin D (imipenem)	PA0958 (*oprD*)	-	-	2.91

* The difference in fold change for antibiotic-resistance gene expression before and after exposure to 1250 µg/mL of NaOCl.

## Data Availability

All data generated or analyzed during this study are included in this published article and its Supplementary Materials. The RNA-Seq and whole-genome sequencing data generated in this study were deposited in the NCBI SRA database under accession code PRJNA1147599.

## Supplementary Materials

The following supporting information can be downloaded at https://www.mdpi.com/article/10.3390/antibiotics13090828/s1. Figure S1: Kyoto Encyclopedia of Genes and Genomes enrichment histogram of *Klebsiella pneumoniae* of genes differentially expressed after exposure to 1250 μg/mL NaOCl; Figure S2: Kyoto Encyclopedia of Genes and Genomes enrichment histogram of *Pseudomonas aeruginosa* of genes differentially expressed after exposure to 1250 μg/mL NaOCl; Table S1 Annotation of draft genome of wide-type and 1250 μg/mL NaOCl-adapted *Klebsiella pneumoniae* and *Pseudomonas aeruginosa*; Table S2: Fold change in expression of genes related to the AcrAB-TolC efflux pump in 1250 μg/mL NaOCl-adapted *Klebsiella pneumoniae*; and Table S3: Results of antibiotic susceptibility tests of 117 strains of Gram-negative bacteria.
