# Supplementary material for: Sublethal Sodium Hypochlorite Exposure: Impact on Resistance-Nodulation-Cell Division Efflux Pump Overexpression and Cross-Resistance to Imipenem
Source: Antibiotics (Basel). 2024 Sep 1;13(9):828. doi: 10.3390/antibiotics13090828 (PMC11429293; doi:10.3390/antibiotics13090828)
Supplement: Supplementary file 1 [file antibiotics-13-00828-s001.zip › antibiotics-3152926-supplementary.pdf]

Supplementary materials

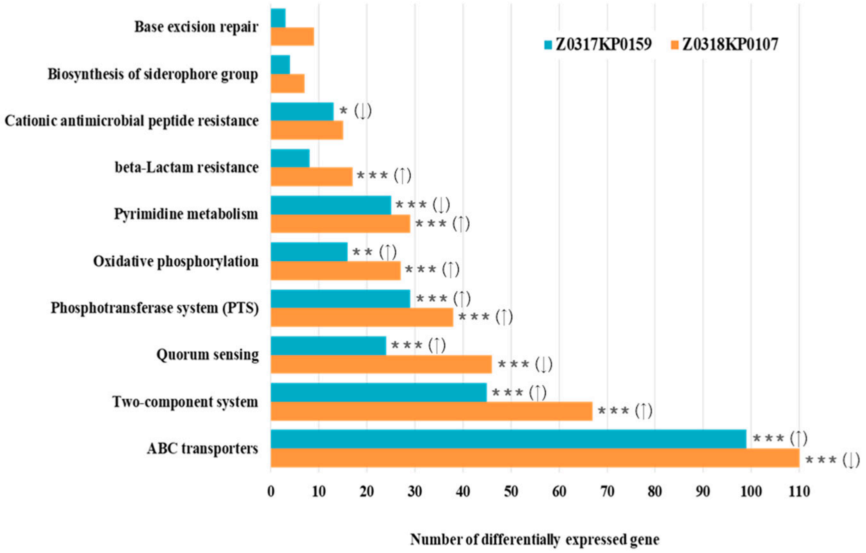

Figure S1. Kyoto Encyclopedia of Genes and Genomes enrichment histogram of *Klebsiella pneumoniae* of genes differentially expressed after exposure to 1,250 μg/ml NaOCl. Statistical significance was determined using modified Fisher's extraction tests with Bonferroni correction ($^{*}P < 0.05$, $^{**}P < 0.01$, $^{***}P < 0.001$).

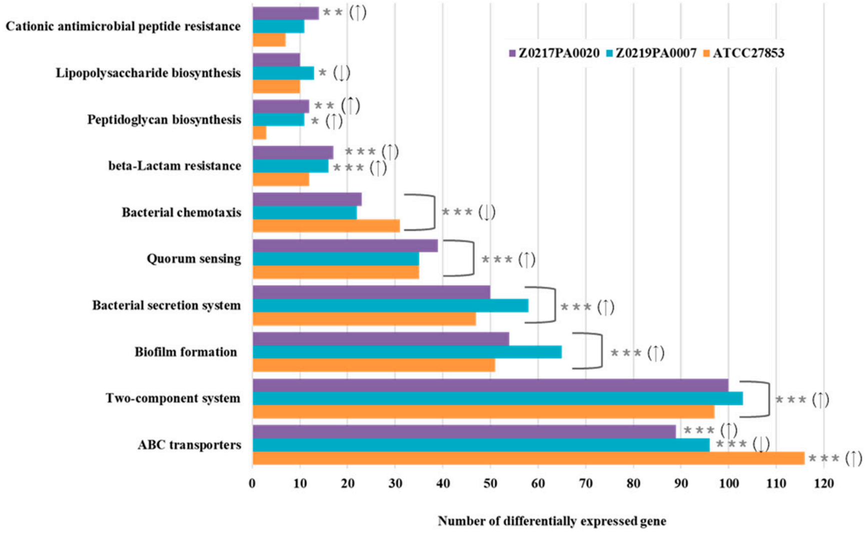

Figure S2. Kyoto Encyclopedia of Genes and Genomes enrichment histogram of *Pseudomonas aeruginosa* of genes differentially expressed after exposure to 1250 μg/ml NaOCl. Statistical significance was determined using modified Fisher's extraction tests with Bonferroni correction ($*P < 0.05$, $**P < 0.01$, $***P < 0.001$).

Table S1. Annotation of draft genome of wide-type and 1,250 μg/ml NaOCl-adapted *Klebsiella pneumoniae* and *Pseudomonas aeruginosa*

		Z0317KP0159		Z0318KP0107		Z2017PA0020		Z0219 PA0007	
		wild	adapted	wild	adapted	wild	adapted	wild	adapted
Chromosome I	length	5,472,227	5,472,256	5,474,465	54,74,991	6,515,133	6,515,216	6,522,343	6,522,024
	CDS	5,039	5,039	5,703	5,704	5,888	5,889	5,896	5,902
	AMG*	bla_{SHV}	bla_{SHV}	bla_{SHV}<br>$bla_{CTX\text{-}M\text{-}1}$	bla_{SHV}<br>$bla_{CTX\text{-}M\text{-}1}$	bla_{PDC}<br>$bla_{OXA\text{-}133}$<br>*cat*<br>*vanW*<br>*tetA*	bla_{PDC}<br>$bla_{OXA\text{-}133}$<br>*cat*<br>*vanW*<br>*tetA*	bla_{PDC}<br>$bla_{OXA\text{-}133}$<br>*cat*<br>*vanW*<br>*tetA*	bla_{PDC}<br>$bla_{OXA\text{-}133}$<br>*cat*<br>*vanW*<br>*tetA*
	DRG**	*qacC*	*qacC*	*qacC*	*qacC*	-	-	-	-
Plasmid I (IncFII)	length	246,731	246,730	246,670	246,694	-	-	-	-
	CDS	262	259	261	258	-	-	-	-
	AMG	bla_{TEM}<br>$bla_{CTX\text{-}M\text{-}1}$<br>$bla_{OXA\text{-}1}$	bla_{TEM}<br>$bla_{CTX\text{-}M\text{-}1}$<br>$bla_{OXA\text{-}1}$	bla_{TEM}<br>$bla_{CTX\text{-}M\text{-}1}$<br>$bla_{OXA\text{-}1}$	bla_{TEM}<br>$bla_{CTX\text{-}M\text{-}1}$<br>$bla_{OXA\text{-}1}$	-	-	-	-
Plasmid II (IncX3)	length	46,836	46,836	46,835	46,836	-	-	-	-
	CDS	59	59	59	59	-	-	-	-
	AMG	bla_{SHV}<br>$bla_{KPC\text{-}2}$	bla_{SHV}<br>$bla_{KPC\text{-}2}$	bla_{SHV}<br>$bla_{KPC\text{-}2}$	bla_{SHV}<br>$bla_{KPC\text{-}2}$	-	-	-	-

* AMG, antimicrobial resistant gene
** DRG, disinfectant resistant gene

Table S2. Fold-change in expression of genes related to the AcrAB-TolC efflux pump in 1,250 μg/ml NaOCl-adapted *Klebsiella pneumoniae*

**Strains**	**Regulator**				**Transporter**		
	acrR	***marR***	***marA***	***soxR***	***acrA***	***acrB***	***tolC***
**Z0317KP0159**	2.4	0.0	8.3	6.8	8.9	2.4	16.4
**Z0318KP0107**	8.9	-6.6	29.1	-49.5	0.0	6.0	22.0

Table S3. Results of antibiotic susceptibility tests of 117 strains of Gram-negative bacteria

Anti-biotics	Gram-negative bacteria (N = 117) *	*E. coli* (n = 48)		*K. pneumoniae* (n = 13)		*A. baumannii* (n = 37)		*P. aeruginosa* (n = 19)	
	**Source**	**Human (n = 41)**	**Env.** ** **(n = 7)**	**Human (n = 9)**	**Env. (n = 4)**	**Human (n = 26)**	**Env. (n = 11)**	**Human (n = 15)**	**Env. (n = 4)**
β-Lactams	Carbapenems	41	0	9	4	26	11	14	0
	Penicillins	40	6	9	4	26	11	8	0
	Cephalosporins	41	5	9	4	26	11	10	2
Aminoglycoside (Amikacin, Gentamicin)		31	1	8	4	26	11	10	0
Fluoroquinolones (Ciprofloxacin)		39	4	9	4	26	11	14	2
Sulfonamides (Trimethoprim-sulfamethoxazole)		34	0	8	2	15	11	-	-
Tetracyclines (Minocycline, Tigecycline)		1	3	1	1	2	0	-	-
Polymyxins (Colistin)		1	0	1	0	8	5	0	0

* With the exception of four reference strains: *Escherichia coli* ATCC 25922 and ATCC 10536, *Acinetobacter baumannii* ATCC 19606, and *Pseudomonas aeruginosa* ATCC 27853.

** Env., environment
